# Lignin Unlocks Stealth Carbon Sinks in Cold Seeps via Microbial Enzymatic Gatekeeping

**DOI:** 10.34133/research.0848

**Published:** 2025-08-25

**Authors:** Jialing Li, Jingchun Feng, Pandeng Wang, Mengzhuo Zhu, Yongji Huang, Ying Wu, Junning Fan, Junlin Hu, Xiyang Dong, Yingli Zhou, Xuanyu Tao, Si Zhang

**Affiliations:** ^1^ Southern Marine Science and Engineering Guangdong Laboratory (Guangzhou), Guangzhou 511458, China.; ^2^ School of Ecology, Environmental, and Resources, Guangdong University of Technology, Guangzhou 510006, China.; ^3^ School of Pharmacy, Shenzhen University Medical School, Shenzhen University, Shenzhen 518055, China.; ^4^ South China Sea Institute of Oceanology, Chinese Academy of Sciences, Guangzhou 510301, China.; ^5^ State Key Laboratory of Estuarine and Coastal Research East China Normal University, Shanghai 200662, China.; ^6^ Key Laboratory of Marine Genetic Resources, Third Institute of Oceanography, Ministry of Natural Resources, Xiamen 361005, China.

## Abstract

Cold seep ecosystems serve as critical hubs in marine carbon cycling through methane emissions and organic matter processing. While terrestrial lignin constitutes a major fraction of persistent organic carbon in cold seep sediments, its microbial transformation pathways in deep-sea cold seep environments remain unexplored. Here, we present the first comprehensive analysis of lignin distribution across sediment horizons at the Haima cold seep, coupled with a multi-omics investigation of microbial lignin metabolism. Laboratory enrichment of sediment communities employing lignin as the exclusive carbon substrate revealed substantial microbial community restructuring dominated by *Burkholderiales*, *Pseudomonadales*, and *Rhizobiales* lineages. Integrated omics resolved 2-tiered metabolic cascades: (a) enzymatic depolymerization via dyP-type peroxidases and LigEFG-mediated β-aryl ether cleavage, targeting syringyl and diarylpropane subunits; (b) funneling of aromatic intermediates through 4,5-/3,4-PDOG (protocatechuate dioxygenase) pathways into central carbon metabolism. Although direct methanogenesis was undetected, methylotrophic potential was evidenced through methane cycle gene expression patterns by lignin decomposers. Phylogenetic surveys further demonstrated the global prevalence of lignin decomposers across 12 major cold seep systems. These decomposers showed marked divergence in enzymatic repertoires compared to degraders from other ecosystems. Our findings establish 3 paradigm shifts: (a) The turnover rates of terrestrial organic carbon are likely underestimated in deep-sea ecosystems; (b) microbial consortia employ combinatorial enzymatic strategies distinct from terrestrial decomposition regimes; (c) methyl shunting from lignin breakdown primes methanogenic precursors, revealing cryptic linkages between refractory carbon cycling and greenhouse gas reservoirs.

## Introduction

Deep-sea cold seeps function as biogeochemical reactors, orchestrating the mineralization and sequestration of organic carbon through tightly coupled microbial processes that shape regional carbon budgets [1–3]. While CH_4_ and CO_2_ represent the terminal phase of carbon mineralization [4,5], the upstream enzymatic machinery governing macromolecular organic carbon breakdown—particularly the catabolism of aromatic polymers—remains a critical blind spot in current carbon flux models [6].

Vertical stratification analyses reveal substantial heterogeneity in organic carbon composition across sediment horizons [7–9], reflecting microbial niche partitioning and substrate specialization. The catabolism of organic carbon through microbial activity constitutes an intricate biochemical network, wherein polymeric macromolecules first undergo enzymatic depolymerization to yield monomeric building blocks, which are then channeled into acidogenesis pathways producing characteristic metabolites including hydrogen gas, short-chain fatty acids (e.g., acetate, propionate, and butyrate), and alcohol derivatives. These intermediates are ultimately metabolized into CH_4_ and CO_2_ [10]. Current paradigms disproportionately emphasize terminal methane cycling, neglecting the rate-limiting initial steps of high-molecular-weight organic carbon processing [11]. This knowledge gap becomes particularly acute when considering lignin as the most abundant terrestrial organic carbon, which accumulates in shelf-margin sediments through terrestrial runoff [12,13].

Lignin is composed of hydroxyphenylpropane monomers linked through various ether and carbon–carbon bonds [14–16], resulting from dehydrogenative polymerization [17]. The initial step in lignin biodegradation involves the action of extracellular lignin-degrading enzymes that catalyze the depolymerization of lignin [18–20]. Microorganisms utilize peroxidases such as laccase and dye-decolorizing peroxidase (DyP) [21,22]; coupled with supplementary enzyme systems, these biocatalysts orchestrate regioselective transformations: cleavage of intermonomeric β-O-4 bonds, separation of alkyl groups from aromatic moieties, methyl group elimination, and disruption of cyclic structures, thereby generating fragmented aromatic molecules. But lignin’s operational dynamics in oxic–suboxic transition zones remain enigmatic in cold seeps.

Previous efforts primarily characterized isolated enzyme systems [23–26], failing to capture how microbial community orchestrates the lignin degradation cascade (macromolecular depolymerization → aromatic monomer conversion → methanogenic precursor formation). This disconnect obscures critical linkages between terrestrial carbon export and marine methane reservoirs. Through laboratory enrichments and in situ analyses, we address 2 fundamental questions: (a) What enzymatic systems enable extracellular lignin modification in suboxic conditions? (b) How do degradation intermediates interface with methane-genic metabolic networks? Here, we employ a tripartite omics framework (metagenomics, metatranscriptomics, and metaproteomics) to dissect lignin transformation pathways in South China Sea cold seep surface sediments.

## Results

### Lignin parameter reveals sustained allochthonous input in Haima cold seep sediments

Quantitative lignin analysis across the Haima cold seep (0 to 25 cm) revealed substantial depleted lignin concentrations [vanillyl (V) phenols: 4.53 to 14.46 μg/g; syringyl (S) phenols: 0.09 to 4.33 μg/g; cinnamyl (C) phenols: 4.46 to 11.47 μg/g] compared to nearshore systems [27–29], establishing cold seeps as distinct lignin reservoirs. Molecular signature analysis (Table S2) identified nonwoody angiosperm sources as the predominant lignin origin (>0 of S/V and C/V ratios), contrasting with terrestrial-dominated coastal inputs. Vertical profiling demonstrated pronounced oxidative modification [P/(S +V): 0.55 to 2.78] across 5 sediment horizons, with the uppermost layer of HOV3 exhibiting relatively lower oxidative degradation and a higher presence of fresh tissues [(Ad/Al) v < 0.3]. This indicates the continuous input and accumulation of lignin in the study area.

### Temporal succession of lignin-responsive microbes reveals niche partitioning in Haima cold seep sediments

**Fig. 1.**
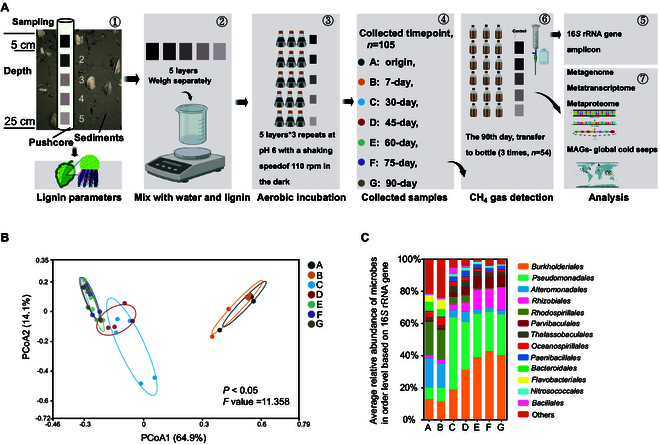
(A) Experimental procedure diagram. The sediments from the Haima cold seep were divided into 5 layers and enriched each with lignin, sampling at 7 intervals; after sampling, a portion of the samples was transferred to hermetically sealed bottles for gas testing of methane content on alternate days. (B) PCoA analysis based on Bray–Curtis distances illustrating the dissimilarities among various samples. (C) Distribution of microbial communities at the order level based on average abundance in different layers (Others <1%). Five layers served as replicates; different time points were analyzed separately in (B) and (C).

Following the enrichment of surface sediment divided into 5 layers using lignin as the exclusive carbon substrate for enrichment (Fig. 1A), the mean residual solid lignin concentration across all microcosms ranged from 2.8 to 4.1 g at day 90. Notably, layer-specific differences in residual lignin were observed: Mean degradation rates were 34.0% ± 9.5% for layer 1, 40.0% ± 4.0% for layer 2, 22.0% ± 4.0% for layer 3, 26.0% ± 4.0% for layer 4, and 34.0% ± 4.0% for layer 5. Furthermore, distinct shifts in microbial communities were observed across a continuous time gradient. The PCoA (principal coordinates analysis) ordination based on Bray–Curtis distances (Fig. 1B) revealed progressive temporal divergence in community structure during enrichment, with trajectories shifting toward assemblages enriched with lignin-degrading taxa (e.g., *Sphingomonadales* and *Burkholderiales*), likely driven by functional adaptation to lignin utilization as the sole carbon source.

The number of core phyla decreased over the course of enrichment (Fig. S1), with microbial communities progressively converging toward core groups potentially capable of lignin utilization. With increasing incubation time, the dominant shared taxa were primarily affiliated with *Gammaproteobacteria*, *Alphaproteobacteria*, and *Bacteroidia* (Fig. S1). Notably, orders such as *Burkholderiales*, *Pseudomonadales*, *Parvibaculales*, and *Rhizobiales* exhibited significant dominance in average relative abundance [Fig. 1C; analysis of variance (ANOVA), *P* < 0.05], and some of these were groups previously reported to be involved in lignin degradation [30–32]. Cluster analysis indicated that the variation in microbial communities along the time gradient was more pronounced than the differences between communities in different layers based on Bray–Curtis distances (Fig. S1). This could be attributed to the increased abundance of potential lignin decomposers.

### Lignin decomposers in Haima cold seep sediments

**Fig. 2.**
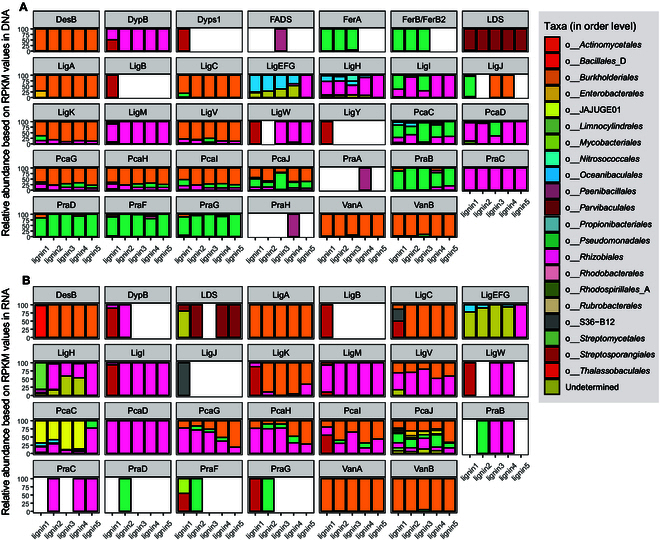
Bacterial communities involved in lignin decomposition. (A) Relative abundance of lignin degradation genes. (B) Transcript abundance of lignin degradation genes. Bars represent the relative abundance of individual decomposing genes. The communities presented here were annotated based on contigs from metagenomes and metatranscriptomes that contain lignin metabolism genes, showing the degradation groups at the order level for microbial community.

Our analysis revealed dynamic lignin degradation patterns in cold seep sediments (Fig. 1 and Fig.S2). While quantitative lignin concentration data were excluded due to evaporation-induced volume variations in shake flask experiments, microbial taxonomic profiling identified key lignin-degrading consortia through functional gene analysis. Phylogenetic reconstruction based on 16*S* ribosomal RNA (rRNA) gene amplicons and metagenome-assembled genomes (MAGs) demonstrated conserved taxonomic patterns of lignin-degrading capacity across 21 bacterial orders, with *Parvibaculales*, *Burkholderiales*, *Pseudomonadales*, and *Rhizobiales* emerging as dominant degraders (Fig. 2A and B).

We observed a degradation change in lignin (Fig. S2). Due to the evaporation of the enriched liquid within the shake flasks, which resulted in significant differences in the liquid volumes inside the flasks, we did not present the lignin concentration. We analyzed microbial taxa possessing genetic components involved in metabolizing lignin and its low-molecular-weight derivatives. This result was similar to that of taxa resulting from 16*S* rRNA gene amplicon. At the order level, decomposers were primarily distributed across 21 orders, with *Parvibaculales*, *Burkholderiales*, *Pseudomonadales*, and *Rhizobiales* being the predominant degrading groups (Fig. 2A and B). The order *Parvibaculales* contains genes for lignin-decomposing DyPs, but due to their low abundance, high-quality MAGs were not obtained in this order; the order *Oceanibaculales* contains genes for glutathione-dependent β-etherases (LigEFG), and the MAGs (bin2, bin24, bin25, and bin29) suggest that *Oceanibaculum nanhaiense* plays a role in lignin degradation at the macromolecular level in the environment (Table S3).

The order *Burkholderiales* primarily participated in the metabolism of syringyl lignin fragments and the 3,4-PDOG pathway. Concurrently, we recovered 4 high-quality bins (bin15, bin22, bin3, and bin60) from the *Burkholderiales* order. These bins were highly abundant across the 5-layer samples and belonged to the genera *Nitrogeniibacter* and *Pusillimonas*, both of which contained genes from these 2 decomposition pathways (Table S3). The order *Pseudomonadales* participated in various steps of lignin fragment decomposition, such as the ferulic acid pathway (FerAB/B_2_) and the 3,4-PDOG pathway, and was also a highly abundant and metabolically active group. We assembled a total of 23 bins belonging to the *Pseudomonadales* order, of which 17 bins contained genes associated with lignin and its degradation, distributed across 8 different genera.

These microorganisms (*Parvibaculales*, *Burkholderiales*, *Pseudomonadales*, and *Rhizobiales*) constituted a relatively high proportion in the South China Sea cold seep ecosystem [1,13,33]. The diverse lignin degraders among them possessed significantly different degradation genes (Fig. S3). These microbial groups serve as pivotal contributors to degradation of lignin macromolecules within the cold seep ecosystem.

### Lignin decomposition pathways in Haima cold seep sediments

**Fig. 3.**
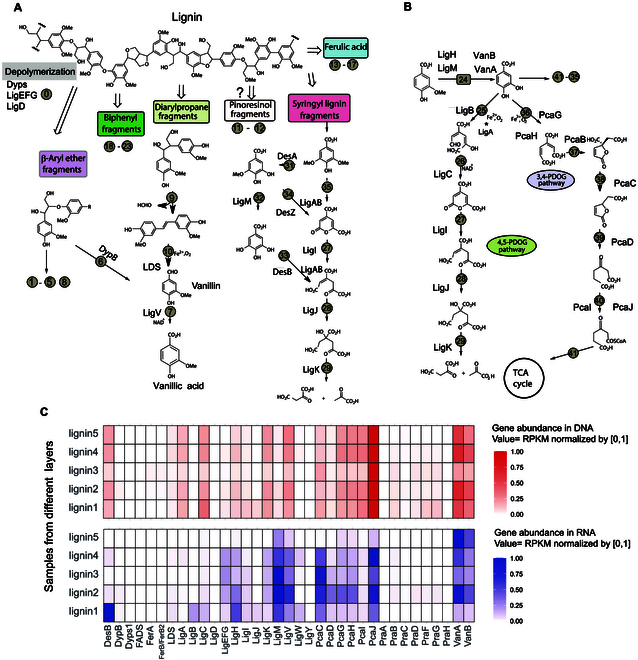
(A) Major degradation pathways of lignin macromolecules in cold seep sediments, with degraders presented at the species level through assembled genomes; key enzymes involved in the degradation process were listed, and Fe^3+^ and O_2_ play roles in electron transfer during lignin degradation. (B) Ring cleavage pathways of lignin-derived aromatic compounds in cold seep sediments. (C) Abundance distribution of these lignin degradation genes in metagenomes and metatranscriptomes, with the *x* axis indicating gene names and the *y* axis representing different sediment layers. Superscript circles represent assigned step numbers for different lignin metabolic pathways. All corresponding gene sources, taxa, functional annotations, and additional details for these numbered steps are comprehensively documented in Table S1.

Reconstruction of lignin and aromatic fragment degradation pathways revealed stratified enzymatic strategies in cold seep sediments (Fig. 3). Genes encoding DyPs and glutathione-dependent β-etherases (LigEFG) were ubiquitously detected across all 5 sediment layers, implicating these enzymes as core mediators of lignin depolymerization. Notably, canonical pathways dependent on multicopper oxidases or Cα-dehydrogenases (LACs) were absent, suggesting niche-specific adaptation of oxidative machinery.

Beyond these depolymerases, multi-omics analysis profiling identified auxiliary oxidoreductases—including catalase, quinone oxidoreductase, aryl alcohol oxidase, and pyranose oxidase—acting as redox partners (Fig. S4 and Table S4) [34–36]. Catalase and quinone oxidoreductase exhibited consistent activity across layers, resolving a critical constraint in this system: While DyPs and lignin peroxidases (LiPs) require H_2_O_2_ to initiate radical-based cleavage [35], its intracellular toxicity necessitates tight regulation via catalase-mediated disproportionation. Concurrently, quinone oxidoreductases likely sustain extracellular redox balance during aromatic fragment processing [35], highlighting layered metabolic cooperation between depolymerization and detoxification modules.

Lignin, after extracellular depolymerization, yields various small molecular aromatic compounds, which are transported into the cell and undergo a series of peripheral catabolic pathways and central metabolic pathways [37]. In this study, lignin small molecular fragments were primarily degraded through 3 pathways: β-aryl ether fragments (⑥ and ⑦), syringyl lignin fragments (㉗ to ㉟), and diarylpropane fragments (⑦, ⑨, and ⑩; Fig. 3A), as the genes for these pathways presented high abundance in the metagenome and metatranscriptome (Fig. 3C). Genes for the biphenyl fragment pathway (LigXYZ) were essentially absent in both the metagenome and metatranscriptome. Genes for the ferulic acid fragments presented at low abundance in the metagenome of the 5-layer samples, but no transcriptional activity was detected across the layers.

Lignin biotransformation proceeds through sequential biochemical phases: initial extracellular depolymerization via oxidative enzymes (e.g., laccases/peroxidases) to yield aromatic oligomers, followed by intracellular funneling of these metabolites where aromatic ring fission proceeds, ultimately generating acetyl-CoA and succinyl-CoA for assimilation into central carbon metabolism. In this study, the degradation of smaller lignin molecule ring cleavage proceeds through the 4,5-PDOG and 3,4-PDOG pathways to the TCA cycle (tricarboxylic acid cycle), with relatively complete gene sets in both the metagenome and metatranscriptome (Fig. 3B). The metaproteomic results also indicate a relatively intact basic metabolic pathway (Fig. S2A).

### Lignin decomposers involved in the broad carbon cycling

We also interrogated the Carbon Cycle Database to demonstrate the broad carbon cycling capabilities of these taxa (Table S4). This additional comparison ensures that our analysis encompasses the wide-ranging potential of these microbial groups in carbon metabolism, which serves as a valuable resource for understanding the role of microorganisms in global carbon cycling.

### Lignin decomposers involved in the methane process

CH_4_ is typically produced in anoxic sediments but can also be produced in oxic conditions [38]. To identify whether lignin-degrading bacteria can utilize intermediate products of lignin to produce methane under microoxic conditions, we transferred the enriched samples cultured for 90 d into sealed bottles and measured the methane production in 5-layer enrichment system. Methane gas production was monitored daily for 3 consecutive days. Methane gas was undetectable throughout the vertically stratified experimental system, maintaining consistency with control group under equivalent analytical protocols (Table S5; ANOVA, *P* < 0.05). We were unable to detect the key methane-producing genes *mcrABG* in enriched samples based on metagenomic (*n* = 5) and transcriptomic data (*n* = 5). This indicates that these lignin-degrading bacteria do not possess the capability to produce methane through the known pathway involving the *mcr* gene.

**Fig. 4.**
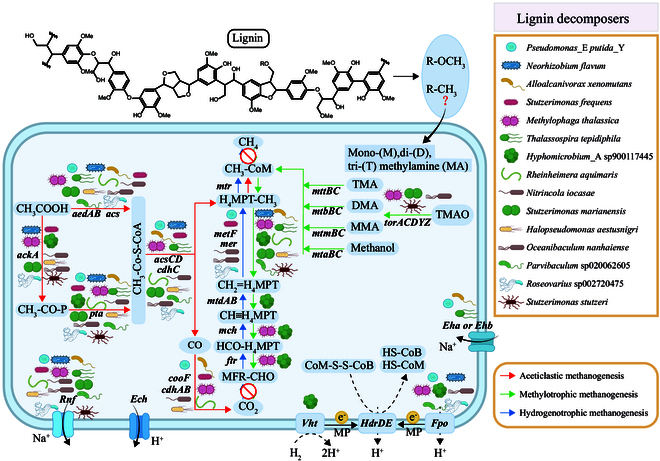
Contribution of lignin degraders to methane production progress. Analysis of methane production genes within lignin-degrading microbial communities reveals their involvement in the methane generation process. It illustrates the annotation of 15 high-quality species-level bins, each containing genes associated with the methane generation process and capable of lignin degradation. The contributions of these species to methane production are indicated adjacent to the corresponding genes in the figure, following the removal of redundant entries. The top panel displays the chemical structure of lignin, and the right side lists the species of microbes involved in lignin degradation.

However, lignin-degrading enzymes facilitate the demethylation of methoxy (-OCH_3_) and methylated (-CH_3_) compounds within lignin [39], leading to the production of methylated small molecular compounds. These small molecules may serve as precursors for methane production in cold seep areas. We have reconstructed a metabolic pathway for methane production by the enriched microbial community (Fig. 4 and Fig. S3). We identified methane production precursor genes associated with lignin-degrading taxa, such as *mtbC* (found in *Rhodobacterales* and *Streptosporangiales*) and *torACZ* genes (present in *Burkholderiales*, *Pseudomonadales*, and *Rhizobiales*) (Fig. 4). The torACZ gene cluster catalyzed the enzymatic conversion of TMAO (trimethylamine-N-oxide) to TMA(trimethylamine) through a reductive biochemical pathway, representing a branch of methylotrophic methane generation [40]. The metaproteomic analysis revealed a considerable abundance of enzymes involved in these methane cycle processes (Fig. S4).

We identified 15 high-quality bins in species level (containing methane generation precursor process genes) with the capability to degrade lignin, after removing repetition. These species’ contributions to the methane generation precursors were annotated next to the related genes in Fig. 4. Unlike the anaerobic methanogenesis pathways in deep sedimentary that utilize nonmethylated precursors, lignin decomposition releases methyl group donors, establishing a unique shallow CH_4_ source with contributions to sediment–water interface methane fluxes.

### Lignin decomposer distribution in global representative cold seeps

**Fig. 5.**
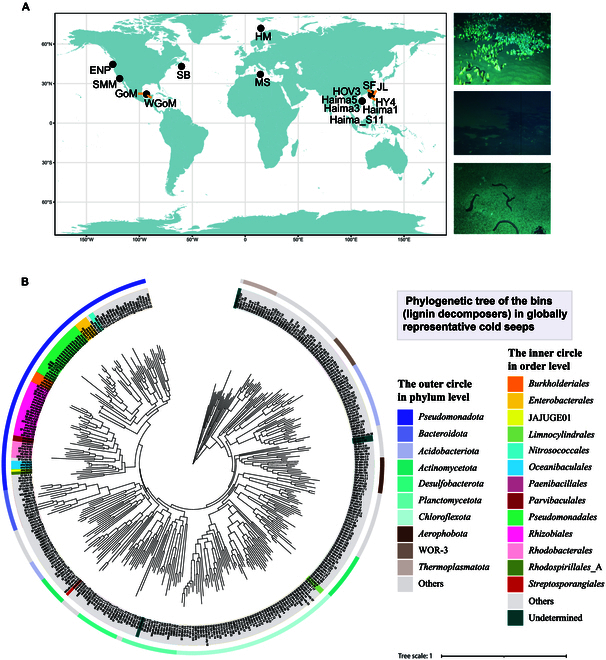
(A) Geographic locations of globally representative cold seeps. (B) Phylogenetic tree of the bins (whole genomes) of lignin-degrading microbial communities in globally representative cold seeps. The outer circle illustrates the distribution of these lignin degraders at the phylum level, with the names of the top 10 most abundant phyla shown; the inner circle shows the distribution of lignin degraders at the order level.

Utilizing a global collection of cold seep metagenomes and metagenomes, we explore lignin-degrading communities based on the 1,495 bins (Table S6). Our findings revealed that 395 of these bins contained genes associated with lignin degradation (Table S7). Constructing a phylogenetic tree with these bins, we observed that these degrading communities were broad distribution within the orders *Burkholderiales*, *Pseudomonadales*, and *Rhizobiales* (Fig. 5B). Genomic analysis revealed a bipartite distribution of lignin-processing capacity: While a limited subset of bins harbored genetic determinants for macromolecular lignin depolymerization, the predominant fraction encoded enzymatic machinery specialized for catabolizing lignin-derived monomers, potentially through cross-pathway recruitment of conserved aromatic degradation orthologs. These degrading communities were distributed across 77 phyla, indicating a broad distribution of lignin degraders in globally representative cold seeps.

## Discussion

Our systematic dissection of lignin processing in the Haima cold seep reveals how microbial consortia govern specific refractory carbon turnover in these cold seep ecosystems. Quantitative sediment profiling identified fresh lignin inputs alongside degradation intermediates, confirming active processing of terrestrial organic matter in marine sediments. Genomically resolved degraders spanning *Burkholderiales*, *Pseudomonadales*, and *Rhizobiales* (Fig. 2) employ complementary oxidative strategies—DyPs and glutathione-dependent β-etherases (LigEFG)—to depolymerize lignin into β-aryl ether, syringyl, and diarylpropane fragments. These products subsequently enter central metabolism via convergent 4,5-PDOG and 3,4-PDOG pathways (Fig. 2A and B), with methylated derivatives potentially subsidizing near-surface methanogenesis through methylotrophic priming (Fig. 4). Crucially, the global prevalence of these degraders across cold seeps [41–44] suggests that lignin processing represents a ubiquitous but overlooked carbon flux in deep-sea cold seeps.

The temporal community divergence may stem from lignin-driven selective pressures that progressively favored specialized taxa through resource partitioning, metabolic adaptation, and cooperative interactions [45,46]. As lignin became the sole carbon source, competitive exclusion shifted communities from generalist heterotrophs (day 0) to lignin-specialized assemblages (days 7, 30, 45, 60, 75, and 90) (Fig. 1B), dominated by taxa (e.g., *Sphingomonadales* and *Burkholderiales*) with enzymatic machinery (e.g., laccases/peroxidases) for cleaving rate-limiting β-aryl ether linkages (Figs. 1C and 2). This metabolic specialization was amplified by in situ enzymatic induction and cross-feeding networks, where primary degraders converted complex lignin into metabolites that sustained secondary utilizers [47], creating syntrophic feedback loops that reinforced the community’s functional adaptation to lignin utilization.

The first step of lignin biodegradation is to depolymerize lignin through extracellular lignin depolymerase. Ligninolytic enzymes employ diverse catalytic mechanisms for polymer decomposition. Laccases (LACs), classified as multicopper oxidoreductases, facilitate lignin depolymerization through radical-mediated oxidation processes mediated by their tetra-nuclear copper cluster [48]. The β-O-4 aryl ether linkages—predominant structural motifs in lignin—undergo specific cleavage through 2 distinct enzymatic systems: NAD^+^-dependent Cα-dehydrogenase (LigD) catalyzes α-carbon oxidation, while the glutathione-dependent LigEFG complex mediates β-ether bond cleavage through nucleophilic thiolysis [49–51]. Furthermore, peroxidase family enzymes demonstrate significant oxidative capacity in lignin modification through hydrogen peroxide-dependent radical chain reactions [46,52]. Previous studies indicate that the most frequently detected lignin-degrading gene families in terrestrial ecosystems include *dypA*, *dypB*, *laCs*, and *mnP*, among others. Terrestrial ecosystems harbor both aerobic and anaerobic lignin degradation pathways, with aerobic pathways being more abundant [53]. In contrast, our study discovered that in the Haima cold seep and globally representative cold seeps, genes encoding enzymes (LigEFG, DyPs) for lignin depolymerization. Degradation proceeds via both aerobic and anaerobic processes, and analysis of enrichment cultures enables the detection of gene signatures associated with anaerobic pathways such as *aor*, *phhB*, and *tyrB* (Fig. S5 and Table S9). We found that a total of 169 bins contained the *phhB* gene, 336 bins contained the *tyrB* gene, and no bins contained the *aor* gene, which is a key gene for the anaerobic process of lignin degradation (Table S10).

In previous estuarine studies, genes such as *lacCs*, *dyPs*, and *ligD* were found to be involved in the depolymerization of lignin [54,55]. In coastal ecosystems, the laccase gene family exhibits high abundance, highlighting its critical role in lignin decomposition within these environments [26,53]. While the abundance of gene families associated with aerobic and anaerobic degradation pathways remains relatively balanced in coastal ecosystems, an increasing trend in the abundance of anaerobic pathway-related gene families has been observed [26,56]. In Haima cold seep, canonical pathways dependent on multicopper oxidases or Cα-dehydrogenases (LACs) were absent, suggesting niche-specific adaptation of oxidative machinery. These findings underscore the intricate variations in lignin depolymerization processes observed among distinct ecosystems. Furthermore, the metabolic routes involved in breaking down lignin-derived aromatic compounds exhibit notable parallels to decomposition pathways documented in estuarine sediments, geothermal wetlands, and terrestrial soil matrices [57–62], with protocatechuic acid being cleaved through 2 distinct routes: the 4,5-PDOG pathway and the 3,4-PDOG pathway.

In terrestrial ecosystems, lignin degradation is primarily mediated by synergistic interactions between fungi and bacteria, with mechanisms exhibiting significant ecosystem specificity [63]. White-rot fungi (e.g., *Phanerochaete chrysosporium*) are the dominant degraders in forest ecosystems, employing secreted ligninolytic enzymes such as LiP, manganese peroxidase (MnP), and laccase to oxidatively dismantle lignin via nonspecific radical reactions that disrupt its aromatic backbone [63,64]. In temperate forests, enzyme activity correlates strongly with climatic conditions; for example, beech forests exhibit concurrent MnP, LiP, and laccase activity, while pine forests rely predominantly on LiP, with degradation rates positively linked to temperature [65]. In contrast, grassland and agricultural systems depend more on bacterial degraders (e.g., *Rhodococcus* spp.), which utilize dioxygenases and demethylases to break down lignin monomers and employ microbial consortia to degrade lignin–cellulose complexes [66]. Notably, the low pH of forest soils favors fungal activity, resulting in higher lignin oxidation rates compared to grasslands and farmlands [66].

In previously studied marine ecosystems, lignin degradation is predominantly bacterial, with *Gammaproteobacteria* (e.g., *Alteromonadaceae* and *Idiomarinaceae*) and *Alphaproteobacteria* excelling in oligotrophic and high-salinity environments through phenylacetyl-CoA (PA-CoA) pathways [67]. Unlike terrestrial systems, marine fungi (e.g., *Ascomycota* and *Basidiomycota*) are scarce, constituting less than 2% of microbial communities [67]. Studies show that marine bacteria achieve lignin mineralization rates of 10% to 20%, relying on dynamic interspecies interactions [68]. In anaerobic sediments, *Bathyarchaeota* contribute to lignin metabolism through demethylation and aromatic ring cleavage, producing acetate for methanogens and forming carbon cycling networks [69,70]. Coastal sediments harbor Proteobacteria and Firmicutes that drive carbon cycling via reconstructed lignin-degrading networks (e.g., β-ketoadipate and protocatechuate branches) [71]. In deep-sea cold seep ecosystems, our analysis based on species-level bins revealed the involvement of certain groups, such as *Methylophaga thalassica*, which had not been previously reported to possess lignin degradation functions. *M. thalassica* carries genes (*praD*, *praF*, *praG*) involved in degrading late-stage lignin-derived small molecules. Global seep analysis identified archaeal bins harboring lignin-degrading genes, mainly including *Asgardarchaeota* and *Thermoplasmatota* (Tables S8 and S10). Notably, no archaeal bins were found to participate in active lignin-utilizing microbial consortia or their associated metabolic pathways under aerobic enrichment conditions (Tables S8 and S10).

In this study, we combined methane gas detection and functional gene analysis, revealing that lignin-degrading bacteria enriched under aerobic conditions lack complete methanogenesis pathways [13,72]. However, these bacteria play a critical role in generating methyl group precursors (e.g., methanol and methylamines) through lignin depolymerization. Under oxygen-limited conditions, isolates like *Klebsiella* sp. LEA1 metabolize lignin into methylated phenols (e.g., 4-vinyl guaiacol) and aromatic acids [73], which may serve as substrates for methylotrophic methanogens. In cold seeps, hydrogen-dependent methylotrophic methanogens (e.g., *Methanomassiliococcales*) utilize these methyl compounds coupled with H₂ [2,4]. Our study identified 15 species-level MAGs encoding key enzymes for methyl transfer (e.g., *mtaB* and *mttC*) and corrinoid cofactor metabolism (Fig. 4), consistent with methylotrophic methanogenesis reported in seep sediments [33]. Persistent deposition of lignocellulose provides sustained carbon input, while microbial redox cycling (e.g., Fe^3+^/Fe^2+^ and S^0^/HS^−^) generates methylotrophic byproducts [74]. This may create anoxic microniches conducive to shallow subsurface methanogenesis near the sediment–water interface. The contribution of this methyl-shunt to CH₄ emissions may be further amplified in shallow zones where methanotrophic activity is suppressed, rendering its impact on sediment-surface CH₄ fluxes non-negligible.

Previous laboratory and natural condition studies have indicated that the degradation period for most microbes targeting lignin was relatively long, ranging from 30 to 60 d, with degradation rates between 20% and 50% [37,46,75,76]. Our cultivation time conditions were conducive to the enrichment of lignin degraders, and changes in the community were detected through PCoA analysis. Taxa such as *Burkholderiales*, *Pseudomonadales*, and *Parvibaculales*, which possess lignin degradation genes, increased significantly over time, demonstrating the effectiveness of our enrichment conditions. We have recovered a group of high-quality bins with lignin degradation capabilities, allowing for a more precise identification of lignin degraders at the species level, which is relatively innovative in the context of cold seep studies. Our laboratory-based experiments could not fully replicate complex in situ conditions, which may limit direct quantitative extrapolation to natural environments. Specifically, the absence of native environmental pressures (e.g., high hydrostatic pressure characteristic of cold seeps) and potential differences in substrate availability/accessibility within the sediment matrix in our enrichment system could alter microbial activity, community dynamics, and, thus, the representativeness of the enriched degraders and their degradation kinetics compared to the true in situ process. While these limitations may affect kinetic rates or absolute degradation efficiencies, our qualitative findings on lignin-degrading consortia and key functional taxa (e.g., dominant bacterial degraders and enzymatic pathways) should remain robust. To address these constraints and improve our understanding of in situ lignin degradation in cold seeps, future studies should employ in situ approaches. This will include deploying high-pressure cultivation systems to simulate the deep-sea conditions, combined with stable isotope probing (SIP) using isotopically labeled lignin substrates. SIP will allow us to directly link metabolic activity to taxonomic identity under relevant pressure conditions, providing a more authentic picture of the key lignin-degrading microorganisms and their functional roles in situ.

## Conclusion

Cold seep ecosystems possess a remarkably robust capacity for lignin degradation. Identifying and confirming the primary microbial pathways for lignin carbon decomposition within these environments is an essential foundational work. Our research has uncovered a suite of cold seep microbial communities with the potential for lignin degradation, which are distributed across taxa such as *Burkholderiales*, *Pseudomonadales*, and *Rhizobiales*. These groups acted on the depolymerization of lignin through genes encoding *dyPs* and *ligEFG*, and subsequently, the breakdown primarily followed 3 pathways: β-aryl ether fragments, syringyl lignin fragments, and diarylpropane fragment. The degradation of smaller lignin molecules then proceeded through the 4,5-PDOG and 3,4-PDOG pathways, entering the TCA cycle. Methylated small-molecule compounds produced from lignin degradation may serve as precursors for methane production in cold seep regions. This evidence the diverse ecological strategies of cold seep microbes and broadens our understanding. The lignin-degrading communities we have identified were widely distributed across global cold seeps and exhibit relatively high abundance, indicating the widespread capacity for lignin degradation among sedimentary microbes in cold seeps.

## Methods

### Sampling in situ

Sediment sampling was conducted at the Haima cold seep site (16°43′N, 110°28′E) located on the northern continental shelf of the South China Sea during April 2023. The collection was performed at a water depth of 1,384 m using a box core sampler with 30-cm penetration depth during the scientific cruise organized by R/V “Kexue”. Seafloor surface sediments were successfully retrieved through standardized box corer deployment procedures, ensuring minimal disturbance to sediment–water interface integrity. Immediately following core retrieval, sediment samples were stabilized through prompt transfer to 4 °C temperature-controlled shipboard storage chambers, where controlled preservation effectively suppressed exogenous microbial metabolisms.

### Incubation experiments

Surface sediments from the cold seep (0 to 25 cm) were collected in 5 stratified layers, with 5 g of each layer mixed with 20 ml of sterilized water to form a slurry. The slurry was equally distributed into conical flasks in triplicate, with 6 ml allocated to each flask. Each flask received 5 g of alkaline lignin (99.9% purity, CAS 8068-05-1; Sigma-Aldrich) as the sole carbon source in a layered cultivation system. Samples were placed into flasks and diluted with sterile water to achieve 150-ml total volume for enrichment culture. Triplicate samples were taken for each layer, yielding a total of 15 treatments. Enrichment conditions were maintained at pH 6 with a shaking speed of 110 rpm in the dark. Samples were collected at 7 time points—0, 7, 30, 45, 60, 75, and 90 d—over a 3-month experimental period. This resulted in a total of 105 samples, with each time point having 5 layers and 3 replicates.

### Lignin parameters

The in situ collected surface sediments (0 to 25 cm) were equally divided into 5 layers, and each layer was measured separately for lignin parameters. After the sediment was naturally air-dried to constant weight, the total organic carbon content of 1-g sediment was determined using a TOC (total organic carbon) analyzer (TOC-VCPN; Shimadzu Corp., Tokyo, Japan). Lignin biomarker quantification was performed following standardized protocols outlined in prior studies [77,78], with method validation through spike recovery tests. The analytical workflow commenced with microwave-assisted alkaline oxidative hydrolysis (165 °C × 3.5 h) using a CEM MARS5 microwave reactor (300 W maximum power). Approximately 2 g of homogenized sediment aliquots was combined in Teflon-lined digestion vessels with reagent-grade CuO, (NH_4_)Fe(SO_4_)_2_ solution, and 2 M NaOH. After undergoing nitrogen sparging, reaction vessels underwent controlled thermal decomposition under inert atmosphere. Post-hydrolysis cooling to ambient temperature facilitated addition of ethyl vanillin as quantitative internal standard, achieving 95% ± 2% mean recovery across triplicate analyses.

Phase separation was achieved through sequential liquid–liquid extraction with ethyl acetate:petroleum ether (99:1 v/v). The combined organic phases were concentrated under N_2_ stream and derivatized with BSTFA [*N*,*O*-bis(trimethylsilyl)tri fluoroacetamide]:TMCS (trimethylchlorosilane) (99:1). Final analysis employed an Agilent 6890N GC-FID system equipped with a DB-1 capillary column, as detailed in [79].

Following incubation, lignin degradation was quantified gravimetrically. After 90 d of treatment, samples were filtered through 0.2-μm membrane filters (Millipore, USA). Lignin retained on the filters was collected, dried overnight at 45 °C in a drying oven, and weighed. The percentage of lignin weight loss was calculated as follows:

$$P_{W}=\left[\left(W_{0}-W_{T}\right)/W_{0}\right]\times 100\%$$ (1)

where *W*_0_ represents the initial lignin weight (g) and *W*_T_ denotes the dry weight (g) at the endpoint (90 d).

All measurements were performed in triplicate.

### CH_4_ flux measurement

On day 90, 15 ml of lignin-enriched suspension samples from the remaining materials was transferred into sealed, light-protected 50-ml glass bottles, with 3 replicates set for each layer and placed in a shaker for cultivation at 25 °C with a rotation speed of 110 rpm. A control was also set up, with the addition of 15 ml of sterilized water. Headspace gas sampling (10 ml/day) was conducted using evacuated aluminized gas sampling bags. To maintain isobaric conditions during gas extraction, a dual-syringe system was implemented: One syringe withdrew gas samples, while a second simultaneously delivered 10 ml of ultrapure helium as pressure compensation. Following sampling, vessels were uncapped and aerated for 30 min under ambient conditions. Methane gas quantification was performed via wavelength-scanned cavity ring-down spectroscopy (WS-CRDS, Picarro G2201-i) with 1-Hz sampling frequency [80]. For 5 treatment groups and 1 control group, samples were collected continuously over 3 d, resulting in a total of 54 gas samples (3 d * 6 treatments * 3 replicates).

### Total protein extraction and detection

Protein extraction from day 90 samples (*n* = 5) was conducted following cryogenic protocols. After ice-thawing, samples were homogenized in BPP buffer (200 mM Tris-Base, 200 mM NaCl, 50 mM vitamin C, 1% sodium deoxycholate, 1% NP-40, 1% Tween 20, 5 mM EDTA, 30% sugar, 50 mM sodium tetraborate, 1% PVPP) using mechanical disruption and centrifuged (12,000*g*, 4 °C, 20 min). Sequential phenol partitioning was performed with tris-saturated phenol under vortexing (10 min, 4 °C), followed by phase separation through successive centrifugations. Proteins were precipitated overnight in ammonium acetate–methanol (−20 °C) and washed twice with 90% acetone. Pellets were resolubilized in denaturing buffer [8 M urea, 1% SDS (sodium dodecyl sulfate), protease inhibitors] with ultrasonic assistance (2-min ice bath). Post-centrifugation supernatants underwent BCA (bicinchoninic acid) quantification (Thermo Scientific kit) against bovine serum albumin standards. Electrophoretic validation was performed using Laemmli SDS–polyacrylamide gel electrophoresis [63] under reducing conditions (150 V, 60 min) [81].

Protein samples (100 μg) underwent sequential processing in TEAB (triethylammonium bicarbonate) buffer: reduction with 10 mM TCEP [Tris(2-carboxyethyl)phosphine; 37 °C, 60 min], cysteine blocking via 40 mM IAM (iodoacetamide; room temperature, dark, 40 min), and clarification by centrifugation (10,000*g*, 4 °C, 20 min). Trypsin digestion (1:50 w/w, 37 °C, 16 h) preceded peptide desalting (HLB cartridges, Oasis HLB 96-well Plate, Waters) after vacuum drying. Reconstituted peptides in 0.1% TFA (trifluoroacetic acid) were quantified spectrophotometrically (NanoDrop One) with external calibration [81].

### Nucleic acid extraction and sequencing

Total nucleic acid from 105 samples was co-isolated via Total RNA Isolation kit (QIAGEN, Germany). RNA was collected from RNA Capture Columns. Retained columns enabled sequential DNA elution (RNA QIAGEN DNA Elution Accessory kit). After merging duplicates, 16*S* rRNA gene amplicons (515F-Y and 926R) from samples (*n* = 35) were sequenced [82]. For the 90th day sampling, a subset underwent metagenome (*n* = 5) and metatranscriptome (*n* = 5) sequencing. Following preprocessing steps, amplicon and metagenome/metatranscriptome sequencing were performed using Illumina NovaSeq 6000 systems (PE250 and PE150) via MAGIGENE (Guangzhou, China), respectively.

### Amplicon analyses

The raw sequencing data were processed as per established protocols [83]. Specifically, paired-end reads were consolidated using USEARCH v11, with primers excised employing CUTADAPT v2.4 software, operationalized with the parameters “--no-indels-e 0 --discard-unrimmed” [84]. Thereafter, low-quality reads defined as length <350 base pairs (bp) or maximum expected error >1.0 were stringently removed. The retained high-quality reads were grouped into amplicon sequence variants (ASVs) using the unoise3 algorithm [85]. Taxonomic assignment of ASVs was conducted with the naïve Bayes classifier [86] through SILVA 138 database analysis in the QIIME2 environment [87]. After removing ASVs unclassified as bacterial or archaeal, a curated ASV table was generated by mapping primer-trimmed reads to the ASVs’ reference sequences, thereby elucidating the microbial taxonomy with precision.

### Metagenomic and metatranscriptomic analyses

Metagenomic sequencing data were subjected to processing in alignment with prior documentation [88]. Low-quality sequences and adapter contaminants were trimmed from raw data using fastp (v0.23.4) [89], followed by scaffold reconstruction of processed reads via SPAdes (v3.15.2) [90]. Open reading frames (ORFs) were computationally predicted for scaffolds >500 bp using Prodigal (v2.6.3) [91]. These ORFs were compiled into a deduplicated gene catalog with CD-HIT-EST (v4.8.1) at 95% identity and 90% coverage thresholds. For metatranscriptomic data, we deployed fastp v0.23.4 to purge low-quality sequences and adapter remnants, and RiboDetector [92] to eliminate rRNA sequences with the parameter “-e rrna”. ORF abundance and transcriptional activity [expressed as reads per kilobase per million mapped reads (RPKM)] were determined by aligning metagenomic/metatranscriptomic reads to the deduplicated gene catalog via Bowtie2 v2.5 [93] and CoverM v0.6.1, with thresholds of 95% identity, 80% read alignment, and 60% coverage. MAGs were extracted from metagenomic datasets and polished using MetaWRAP [94]. MAG quality was evaluated via CheckM v1.0.12 [95], while PROKKA v1.12 annotated MAGs meeting high-quality criteria (>60% completeness, <5% contamination) [96].

### Reconstruction of lignin decomposition pathway

We constructed a lignin database comprising a comprehensive collection of lignolytic enzyme sequences, curated from published literature [53,97]. The deduplicated gene dataset was screened against these reference proteins using DIAMOND v0.9 with thresholds “--id 50 --query-cover 80” [98]. Cumulative RPKM values for ORFs encoding target enzymes were calculated to quantify ligninolytic potential. Gene expression patterns were analyzed to estimate pathway activation status. Bacterial genomes (MAGs) associated with lignin degradation were identified through protein sequence comparison against the reference database using DIAMOND v0.9. Genomic assignments to specific metabolic pathways were determined through systematic evaluation of enzymatic gene inventories within each MAG. To determine taxonomic assignments of ligninolytic-associated genes, alignments were performed against the GTDB r95 proteome database using DIAMOND v0.9 with “—evalue 0.00001 -k 10” parameters. Taxonomic classification for each gene was established by applying the Last Common Ancestor (LCA) algorithm to the 10 highest-ranking matches. Taxonomic consistency between databases was ensured by converting GTDB classifications to National Center for Biotechnology Information (NCBI) taxonomy through the GTDB-provided mapping file.

Additionally, we employed the LCdb database to identify lignin-degrading genes from deduplicated gene dataset. Protein sequences were aligned against the LCdb reference database using DIAMOND v2.0.14. Then, alignment results were functionally annotated using a custom Python script. To investigate the distribution of lignin-degrading genes in global cold seep ecosystems, we analyzed MAGs identified through protein sequence comparison against the LCdb database [53].

### Methane metabolic pathway

We downloaded the carbon cycling database CcycDB (https://ccycdb.github.io/) and the methane cycling database McycDB (https://github.com/qichao1984/). Subsequently, using DIAMOND v0.9 with parameters set as described in the literature [40], we interrogated the nonredundant gene catalog obtained above against the sequences in these databases. Gene expression patterns of these target genes were analyzed to assess corresponding pathway activation states. Furthermore, MAG-derived ORFs were identified through database sequence alignment. Systematic evaluation of enzymatic gene inventories enabled precise mapping of MAGs to methane metabolism pathways.

### Metaproteomic identification and analyses

For the data-independent acquisition (DIA) raw data analysis, Spectronaut software (Version 18) was utilized [99]. Quantitative profiling utilized 6 peptides per protein with 3 fragment ions per peptide, under the following parameters: protein false discovery rate (FDR) cutoff ≤0.01%, peptide FDR ≤0.01%, identification confidence ≥99%, and XIC mass tolerance ≤75 parts per million [99]. Peptides that were shared or modified were excluded from the analysis. Quantification results were derived through integration and summation of chromatographic peak intensities [100]. Protein identification was conducted solely for those proteins possessing at least one unique peptide. Proteomic data processing was conducted via the Majorbio Cloud Platform (https://cloud.majorbio.com), followed by metagenome-informed functional annotation of all detected proteins [101].

### Lignin decomposers in representative global cold seeps

Metagenomic assemblies (MAGs) derived from 68 sediment specimens obtained from 6 globally representative cold seep ecosystems were retrieved from public repositories [7,41,72]. Sediment cores encompassed vertical redox zonation across 430-cm subseafloor depth, transitioning from oxic surficial zones to sulfidic strata at the sediment–water interface. These MAG data were species-delineated based on a 95% average nucleotide identity threshold, recovering a total of 1,427 species-level clusters. In combination with the 68 bins from our lignin-enriched samples, we obtained 1,495 species-level clusters in total (1,214 bacteria and 281 archaea; Table S6), which were discerned against a lignin database. Metagenomic bins harboring genetic determinants for lignin metabolism were subjected to phylogenetic reconstruction through maximum likelihood analysis implemented in the Phylophlan 3.0 platform (v3.7.1) [102]. Subsequent topological visualization of the refined phylogenetic tree (RAxML_bestTree.refined.tre) was executed using the Interactive Tree of Life interface (iTOL v6.7.3) [103].

### Statistical analysis

Statistical evaluation of lignin composition (components, sources, and characteristics), temporal stratification effects on microbiome community, and methane accumulation gas was performed using one-way ANOVA. The computational framework incorporated customized analytical pipelines executed in R version 4.2.2, leveraging both base statistical functions and specialized ecological analysis modules from the vegan package (v2.6-4) for comprehensive multivariate comparisons across 5 distinct sedimentary horizons.

## Data Availability

Multi-omics sequencing datasets (16*S* rRNA gene amplicon sequencing, metagenomics, and metatranscriptomics) have been archived in the NCBI Sequence Read Archive repository under BioProject accessions PRJNA1205479-PRJNA1206677. Complementary experimental datasets supporting the conclusions of this work are maintained by the corresponding investigator team and can be accessed through formal data request protocols.

## References

[1] Jiang Q, Jing H, Jiang Q, Zhang Y. Insights into carbon-fixation pathways through metagonomics in the sediments of deep-sea cold seeps. Mar Pollut Bull. 2022;176: Article 113458. 35217425 10.1016/j.marpolbul.2022.113458

[2] Lv Y, Yang S, Xiao X, Zhang Y. Stimulated organic carbon cycling and microbial community shift driven by a simulated cold-seep eruption. MBio. 2022;13(2): Article e0008722. 35229641 10.1128/mbio.00087-22 PMC8941925

[3] Yang S, Lv Y, Liu X, Wang Y, Fan Q, Yang Z, Boon N, Wang F, Xiao X, Zhang Y. Genomic and enzymatic evidence of acetogenesis by anaerobic methanotrophic archaea. Nat Commun. 2020;11(1): Article 3941. 32770005 10.1038/s41467-020-17860-8 PMC7414198

[4] Boetius A, Wenzhöfer F. Seafloor oxygen consumption fuelled by methane from cold seeps. Nat Geosci. 2013;6(9):725–734.

[5] Jiang Q, Jing H, Liu H, Du M. Biogeographic distributions of microbial communities associated with anaerobic methane oxidation in the surface sediments of deep-sea cold seeps in the South China Sea. Front Microbiol. 2022;13: Article 1060206. 36620029 10.3389/fmicb.2022.1060206 PMC9822730

[6] Feng J, Yan J, Wang Y, Yang Z, Zhang S, Liang S, Li X. Methane mitigation: Learning from the natural marine environment. Innovation. 2022;3(5): Article 100297. 10.1016/j.xinn.2022.100297 PMC9425072 36051819

[7] Dong X, Peng Y, Wang M, Woods L, Wu W, Wang Y, Xiao X, Li J, Jia K, Greening C, et al. Evolutionary ecology of microbial populations inhabiting deep sea sediments associated with cold seeps. Nat Commun. 2023;14(1): Article 1127. 36854684 10.1038/s41467-023-36877-3 PMC9974965

[8] Wu X, Feng J, Chen X, Li C, Zhang S. Exploring carbon content variation in microplastics sequestrated from seawater to sediment in the Haima cold seep area. J Hazard Mater. 2024;462: Article 132742. 37871440 10.1016/j.jhazmat.2023.132742

[9] Zhang X, Du Z, Zheng R, Luan Z, Qi F, Cheng K, Wang B, Ye W, Liu X, Lian C, et al. Development of a new deep-sea hybrid Raman insertion probe and its application to the geochemistry of hydrothermal vent and cold seep fluids. Deep-Sea Res Pt I Oceanogr Res Pap. 2017;123:1–12.

[10] Zhai X, Shi X, Cheng H, Yao P, Zhao B, Chen L, Liu J, Cao L, Wang M, Fu L, et al. Horizontal and vertical heterogeneity of sediment microbial community in Site F cold seep, the South China Sea. Front Mar Sci. 2022;9: Article 957762.

[11] Muyzer G, Stams AJM. The ecology and biotechnology of sulphate-reducing bacteria. Nat Rev Microbiol. 2008;6(6):441–454. 18461075 10.1038/nrmicro1892

[12] Bhattarai S, Cassarini C, Lens PNL. Physiology and distribution of archaeal methanotrophs that couple anaerobic oxidation of methane with sulfate reduction. Microbiol Mol Biol Rev. 2019;83(3): Article e00074-18. 10.1128/MMBR.00074-18 PMC6710461 31366606

[13] Feng D, Qiu J, Hu Y, Peckmann J, Guan H, Tong H, Chen C, Chen J, Gong S, Li N, et al. Cold seep systems in the South China Sea: An overview. J Asian Earth Sci. 2018;168:3–16.

[14] Kandasamy S, Nath BN. Perspectives on the terrestrial organic matter transport and burial along the land-deep sea continuum: Caveats in our understanding of biogeochemical processes and future needs. Front Mar Sci. 2016;3: Article 00259.

[15] Schlunz B, Schneider RR. Transport of terrestrial organic carbon to the oceans by rivers: Re-estimating flux- and burial rates. Int J Earth Sci. 2000;88(4):599–606.

[16] Yu S, Zhou Y, Gan M, Chen L, Xie Y, Zhong Y, Feng Q, Chen C. Lignocellulose-based optical biofilter with high near-infrared transmittance via lignin capturing–fusing approach. Research. 2023;6: Article 0250. 37869743 10.34133/research.0250 PMC10585486

[17] Kamimura N, Sakamoto S, Mitsuda N, Masai E, Kajita S. Advances in microbial lignin degradation and its applications. Curr Opin Biotechnol. 2019;56:179–186. 30530243 10.1016/j.copbio.2018.11.011

[18] Chen Z, Wan C. Biological valorization strategies for converting lignin into fuels and chemicals. Renew Sust Energ Rev. 2017;73:610–621.

[19] Lou Y, Sun X, Yu Y, Zeng S, Li Y, Liu Y, Yu H. One-pot protolignin extraction by targeted unlocking lignin–carbohydrate esters via nucleophilic addition–elimination strategy. Research. 2023;6: Article 0069. 36930767 10.34133/research.0069 PMC10013968

[20] Qu X, Zhao Y, Za C, Wang S, Ren Y, Wang Q, Shao J, Wang W, Dong X. Thermoresponsive lignin-reinforced poly(ionic liquid) hydrogel wireless strain sensor. Research. 2021;2021: Article 9845482. 34957404 10.34133/2021/9845482 PMC8674648

[21] Palazzolo MA, Kurina-Sanz M. Microbial utilization of lignin: Available biotechnologies for its degradation and valorization. World J Microbiol Biotechnol. 2016;32(10): Article 173. 27565783 10.1007/s11274-016-2128-y

[22] Abdelaziz OY, Brink DP, Prothmann J, Ravi K, Sun M, García-Hidalgo J, Sandahl M, Hulteberg CP, Turner C, Lidén G, et al. Biological valorization of low molecular weight lignin. Biotechnol Adv. 2016;34(8):1318–1346. 27720980 10.1016/j.biotechadv.2016.10.001

[23] Bonin P, Portas A, Hardy J, Guasco S, Bianchi TS, Ward ND, Rontani J-F. Biological mechanisms underlying priming of vascular plant material in the presence of diatoms. Aquat Microb Ecol. 2023;89:99–117.

[24] Dang Y, Cha Q, Liu S, Wang S, Li P, Li C, Wang P, Chen X, Tian J, Xin Y, et al. Phytoplankton-derived polysaccharides and microbial peptidoglycans are key nutrients for deep-sea microbes in the Mariana Trench. Microbiome. 2024;12(1): Article 77. 38664737 10.1186/s40168-024-01789-x PMC11044484

[25] Leadbeater DR, Oates NC, Bennett JP, Li Y, Dowle AA, Taylor JD, Alponti JS, Setchfield AT, Alessi AM, Helgason T, et al. Mechanistic strategies of microbial communities regulating lignocellulose deconstruction in a UK salt marsh. Microbiome. 2021;9(1): Article 48. 10.1186/s40168-020-00964-0 PMC7890819 33597033

[26] Peng Q, Lin L, Tu Q, Wang X, Zhou Y, Chen J, Jiao N, Zhou J. Unraveling the roles of coastal bacterial consortia in degradation of various lignocellulosic substrates. mSystems. 2023;8(4): Article e0128322. 37417747 10.1128/msystems.01283-22 PMC10469889

[27] Bao H, Wu Y, Zhan X, Wang X, Spencer RGM, Hernes PJJ, Feng X, Lee L, Huang J, Zhang J, et al. Global riverine export of dissolved lignin constrained by hydrology, geomorphology, and land-cover. Glob Biogeochem Cycles. 2023;37(4): Article e2022GB007607.

[28] Spencer RGM, Stubbins A, Hernes PJ, Baker A, Mopper K, Aufdenkampe AK, Dyda RY, Mwamba VL, Mangangu AM, Wabakanghanzi JN, et al. Photochemical degradation of dissolved organic matter and dissolved lignin phenols from the Congo River. J Geophys Res Biogeosci. 2009;114(G3): Article 2009JG000968.

[29] Dittmar T, Hertkorn N, Kattner G, Lara RJ. Mangroves, a major source of dissolved organic carbon to the oceans. Glob Biogeochem Cycles. 2006;20(1): Article 2005GB002570.

[30] Ahmad M, Taylor CR, Pink D, Burton K, Eastwood D, Bending GD, Bugg TD. Development of novel assays for lignin degradation: Comparative analysis of bacterial and fungal lignin degraders. Mol BioSyst. 2010;6(5):815–821. 20567767 10.1039/b908966g

[31] Brink DP, Ravi K, Lidén G, Gorwa-Grauslund MF. Mapping the diversity of microbial lignin catabolism: Experiences from the eLignin database. Appl Microbiol Biotechnol. 2019;103(10):3979–4002. 30963208 10.1007/s00253-019-09692-4 PMC6486533

[32] Bugg TD, Ahmad M, Hardiman EM, Rahmanpour R. Pathways for degradation of lignin in bacteria and fungi. Nat Prod Rep. 2011;28(12):1883–1896. 21918777 10.1039/c1np00042j

[33] Zhang H, Wang M, Wang H, Chen H, Cao L, Zhong Z, Lian C, Zhou L, Li C. Metagenome sequencing and 768 microbial genomes from cold seep in South China Sea. Sci Data. 2022;9(1): Article 480. 35933411 10.1038/s41597-022-01586-x PMC9357000

[34] Brown ME, Walker MC, Nakashige TG, Iavarone AT, Chang MCY. Discovery and characterization of heme enzymes from unsequenced bacteria: Application to microbial lignin degradation. J Am Chem Soc. 2011;133(45):18006–18009. 21671563 10.1021/ja203972q

[35] Yamada Y, Wang J, Kawagishi H, Hirai H. Improvement of ligninolytic properties by recombinant expression of glyoxal oxidase gene in hyper lignin-degrading fungus Phanerochaete sordida YK-624. Biosci Biotechnol Biochem. 2014;78(12):2128–2133. 25117933 10.1080/09168451.2014.946398

[36] Ma J, Zhang K, Liao H, Hector SB, Shi X, Li J, Liu B, Xu T, Tong C, Liu X, et al. Genomic and secretomic insight into lignocellulolytic system of an endophytic bacterium Pantoea ananatis Sd-1. Biotechnol Biofuels. 2016;9(1): Article 25. 10.1186/s13068-016-0439-8 PMC4736469 26839588

[37] Atiwesh G, Parrish CC, Banoub J, Le TT. Lignin degradation by microorganisms: A review. Biotechnol Prog. 2022;38(2): Article e3226. 34854261 10.1002/btpr.3226

[38] Peeters F, Hofmann H. Oxic methanogenesis is only a minor source of lake-wide diffusive CH_4_ emissions from lakes. Nat Commun. 2021;12(1): Article 1206. 10.1038/s41467-021-21215-2 PMC7900097 33619253

[39] Mallinson SJB, Machovina MM, Silveira RL, Garcia-Borràs M, Gallup N, Johnson CW, Allen MD, Skaf MS, Crowley MF, Neidle EL, et al. A promiscuous cytochrome P450 aromatic O-demethylase for lignin bioconversion. Nat Commun. 2018;9(1): Article 2487. 29950589 10.1038/s41467-018-04878-2 PMC6021390

[40] Qian L, Yu X, Zhou J, Gu H, Ding J, Peng Y, He Q, Tian Y, Liu J, Wang S, et al. MCycDB: A curated database for comprehensively profiling methane cycling processes of environmental microbiomes. Mol Ecol Resour. 2022;22(5):1803–1823. 35080120 10.1111/1755-0998.13589

[41] Dong X, Rattray JE, Campbell DC, Webb J, Chakraborty A, Adebayo O, Matthews S, Li C, Fowler M, Morrison NM, et al. Thermogenic hydrocarbon biodegradation by diverse depth-stratified microbial populations at a Scotian Basin cold seep. Nat Commun. 2020;11(1): Article 5825. 33203858 10.1038/s41467-020-19648-2 PMC7673041

[42] Han Y, Zhang C, Zhao Z, Peng Y, Liao J, Jiang Q, Liu Q, Shao Z, Dong X. A comprehensive genomic catalog from global cold seeps. Sci Data. 2023;10(1): Article 596. 37684262 10.1038/s41597-023-02521-4 PMC10491686

[43] Quan Q, Liu J, Li C, Ke Z, Tan Y. Insights into prokaryotic communities and their potential functions in biogeochemical cycles in cold seep. mSphere. 2024;9(10): Article e0054924. 39269181 10.1128/msphere.00549-24 PMC11524163

[44] Chen J, Jia Y, Sun Y, Liu K, Zhou C, Liu C, Li D, Liu G, Zhang C, Yang T, et al. Global marine microbial diversity and its potential in bioprospecting. Nature. 2024;633(8029):371–379. 39232160 10.1038/s41586-024-07891-2 PMC11390488

[45] Levy-Booth DJ, Navas LE, Fetherolf MM, Liu LY, Dalhuisen T, Renneckar S, Eltis LD, Mohn WW. Discovery of lignin-transforming bacteria and enzymes in thermophilic environments using stable isotope probing. ISME J. 2022;16(8):1944–1956. 35501417 10.1038/s41396-022-01241-8 PMC9296663

[46] Zhao L, Zhang J, Zhao D, Jia L, Qin B, Cao X, Zang L, Lu F, Liu F. Biological degradation of lignin: A critical review on progress and perspectives. Ind Crop Prod. 2022;188: Article 115715.

[47] Zhang R, Wang J, Milligan S, Yan Y. Microbial utilization of lignin-derived aromatics via a synthetic catechol meta-cleavage pathway. Green Chem. 2021;23(20):8238–8250.

[48] Sirim D, Wagner F, Wang L, Schmid RD, Pleiss J. The laccase engineering database: A classification and analysis system for laccases and related multicopper oxidases. Database. 2011;2011: Article bar006. 21498547 10.1093/database/bar006 PMC3077825

[49] Wang X, Lin L, Zhou J. Links among extracellular enzymes, lignin degradation and cell growth establish the models to identify marine lignin-utilizing bacteria. Environ Microbiol. 2021;23(1):160–173. 33107668 10.1111/1462-2920.15289

[50] Marinović M, Nousiainen P, Dilokpimol A, Kontro J, Moore R, Sipilä J, de Vries RP, Mäkelä MR, Hildén K. Selective cleavage of lignin β-O-4 aryl ether bond by β-etherase of the white-rot fungus Dichomitus squalens. ACS Sustain Chem Eng. 2018;6(3):2878–2882. 30271687 10.1021/acssuschemeng.7b03619 PMC6156110

[51] Pereira JH, Heins RA, Gall DL, McAndrew RP, Deng K, Holland KC, Donohue TJ, Noguera DR, Simmons BA, Sale KL, et al. Structural and biochemical characterization of the early and late enzymes in the lignin β-aryl ether cleavage pathway from Sphingobium sp. SYK-6*. J Biol Chem. 2016;291(19):10228–10238. 26940872 10.1074/jbc.M115.700427 PMC4858972

[52] Hendel B, Sinsabaugh RL, Marxsen J. Lignin-degrading enzymes: Phenoloxidase and peroxidase. In: Bärlocher F, Gessner MO, Graça MAS, editors. *Methods to study litter decomposition: A practical guide*. Cham: Springer International Publishing; 2020. p. 425–431.

[53] Chen J, Lin L, Tu Q, Peng Q, Wang X, Liang C, Zhou J, Yu X. Metagenomic-based discovery and comparison of the lignin degrading potential of microbiomes in aquatic and terrestrial ecosystems via the LCdb database. Mol Ecol Resour. 2024;24(5): Article e13950. 38567644 10.1111/1755-0998.13950

[54] Dittmar T, Lara RJ. Molecular evidence for lignin degradation in sulfate-reducing mangrove sediments (Amazônia, Brazil). Geochim Cosmochim Acta. 2001;65(9):1417–1428.

[55] Li J, Duan L, Wu Y, Ahmad M, Yin L, Luo X, Wang X, Fang B, Li S, Huang L, et al. Unraveling microbe-mediated degradation of lignin and lignin-derived aromatic fragments in the Pearl River Estuary sediments. Chemosphere. 2022;296: Article 133995. 35176304 10.1016/j.chemosphere.2022.133995

[56] Ma W, Lin L, Peng Q. Origin, selection, and succession of coastal intertidal zone-derived bacterial communities associated with the degradation of various lignocellulose substrates. Microb Ecol. 2023;86(3):1589–1603. 36717391 10.1007/s00248-023-02170-5

[57] Levy-Booth DJ, Hashimi A, Roccor R, Liu L-Y, Renneckar S, Eltis LD, Mohn WW. Genomics and metatranscriptomics of biogeochemical cycling and degradation of lignin-derived aromatic compounds in thermal swamp sediment. ISME J. 2021;15(3):879–893. 33139871 10.1038/s41396-020-00820-x PMC8027834

[58] Ferrer A, Heath KD, Canam T, Flores HD, Dalling JW. Contribution of fungal and invertebrate communities to wood decay in tropical terrestrial and aquatic habitats. Ecology. 2020;101(9): Article e03097. 32415862 10.1002/ecy.3097

[59] Chen J, Luo Y, García-Palacios P, Cao J, Dacal M, Zhou X, Li J, Xia J, Niu S, Yang H, et al. Differential responses of carbon-degrading enzyme activities to warming: Implications for soil respiration. Glob Chang Biol. 2018;24(10):4816–4826. 29999577 10.1111/gcb.14394

[60] Woo HL, Hazen TC, Simmons BA, DeAngelis KM. Enzyme activities of aerobic lignocellulolytic bacteria isolated from wet tropical forest soils. Syst Appl Microbiol. 2014;37(1):60–67. 24238986 10.1016/j.syapm.2013.10.001

[61] Wilhelm RC, Singh R, Eltis LD, Mohn WW. Bacterial contributions to delignification and lignocellulose degradation in forest soils with metagenomic and quantitative stable isotope probing. ISME J. 2019;13(2):413–429. 30258172 10.1038/s41396-018-0279-6 PMC6331573

[62] Janvier M, Grimont PA. The genus Methylophaga, a new line of descent within phylogenetic branch gamma of Proteobacteria. Res Microbiol. 1995;146(7):543–550. 8577995 10.1016/0923-2508(96)80560-2

[63] Fujii K, Nakada Y, Umezawa K, Yoshida M, Shibata M, Hayakawa C, Inagaki Y, Kosaki T, Hangs R. A comparison of lignin-degrading enzyme activities in forest floor layers across a global climatic gradient. Soil Ecol Lett. 2020;2(4):281–294.

[64] Boyle CD, Kropp BR, Reid ID. Solubilization and mineralization of lignin by white rot fungi. Appl Environ Microbiol. 1992;58(10):3217–3224. 16348781 10.1128/aem.58.10.3217-3224.1992 PMC183083

[65] Chio C, Sain M, Qin W. Lignin utilization: A review of lignin depolymerization from various aspects. Renew Sust Energ Rev. 2019;107:232–249.

[66] Angst G, Mueller KE, Nierop KGJ, Simpson MJ. Plant- or microbial-derived? A review on the molecular composition of stabilized soil organic matter. Soil Biol Biochem. 2021;156: Article 108189.

[67] Su J, Li D, Chen Q, Li M, Su L, Luo T, Liang D, Lai G, Shuai O, Jiao C, et al. Anti-breast cancer enhancement of a polysaccharide from spore of Ganoderma lucidum with paclitaxel: Suppression on tumor metabolism with gut microbiota reshaping. Front Microbiol. 2018;9: Article 3099. 30619178 10.3389/fmicb.2018.03099 PMC6304348

[68] Peng Q, Zhao C, Wang X, Cheng K, Wang C, Xu X, Lin L. Modeling bacterial interactions uncovers the importance of outliers in the coastal lignin-degrading consortium. Nat Commun. 2025;16(1): Article 639. 39809803 10.1038/s41467-025-56012-8 PMC11733112

[69] Yu T, Wu W, Liang W, Wang Y, Hou J, Chen Y, Elvert M, Hinrichs K-U, Wang F. Anaerobic degradation of organic carbon supports uncultured microbial populations in estuarine sediments. Microbiome. 2023;11(1): Article 81. 37081504 10.1186/s40168-023-01531-z PMC10116835

[70] Yu T, Hu H, Zeng X, Wang Y, Pan D, Deng L, Liang L, Hou J, Wang F. Widespread Bathyarchaeia encode a novel methyltransferase utilizing lignin-derived aromatics. mLife. 2023;2(3):272–282. 38817817 10.1002/mlf2.12082 PMC10989822

[71] Ley Y, Cheng X-Y, Ying Z-Y, Zhou N-Y, Xu Y. Characterization of two marine lignin-degrading consortia and the potential microbial lignin degradation network in nearshore regions. Microbiol Spectr. 2023;11(3): Article e0442422. 37042774 10.1128/spectrum.04424-22 PMC10269927

[72] Dong X, Zhang C, Peng Y, Zhang H, Shi L, Wei G, Hubert CRJ, Wang Y, Greening C. Phylogenetically and catabolically diverse diazotrophs reside in deep-sea cold seep sediments. Nat Commun. 2022;13(1): Article 4885. 35985998 10.1038/s41467-022-32503-w PMC9391474

[73] Sumranwanich T, Amosu E, Chankhamhaengdecha S, Phetruen T, Loktumraks W, Ounjai P, Harnvoravongchai P. Evaluating lignin degradation under limited oxygen conditions by bacterial isolates from forest soil. Sci Rep. 2024;14(1): Article 13350. 38858437 10.1038/s41598-024-64237-8 PMC11164938

[74] Bhattarai S, Cassarini C, Rene ER, Kummel S, Esposito G, Lens P. Enrichment of ANME-2 dominated anaerobic oxidation of methane coupled to sulfate reduction consortia from cold seep sediment (Ginsburg Mud Volcano, Gulf of Cadiz) in a membrane bioreactor. In: *Performance assessment and enrichment of anaerobic methane oxidizing microbial communities from marine sediments in bioreactors*. London (UK): CRC Press; 2018. p. 369–378. .

[75] Tuomela M, Vikman M, Hatakka A, Itävaara M. Biodegradation of lignin in a compost environment: A review. Bioresour Technol. 2000;72(2):169–183.

[76] Thevenot M, Dignac M-F, Rumpel C. Fate of lignins in soils: A review. Soil Biol Biochem. 2010;42(8):1200–1211.

[77] Hedges JI, Mann DC. The characterization of plant tissues by their lignin oxidation products. Geochim Cosmochim Acta. 1979;43(11):1803–1807.

[78] Goñi MA, Montgomery S. Alkaline CuO oxidation with a microwave digestion system: Lignin analyses of geochemical samples. Anal Chem. 2000;72(14):3116–3121. 10939375 10.1021/ac991316w

[79] Yu H, Wu Y, Zhang J, Deng B, Zhu Z. Impact of extreme drought and the Three Gorges Dam on transport of particulate terrestrial organic carbon in the Changjiang (Yangtze), River. J Geophys Res Earth. 2011;116(F4): Article 2011JF002012.

[80] Li X, Yao H, Yu Y, Cao Y, Tang C. Greenhouse gases in an urban river: Trend, isotopic evidence for underlying processes, and the impact of in-river structures. J Hydrol. 2020;591: Article 125290.

[81] Xu M, Deng J, Xu K, Zhu T, Han L, Yan Y, Yao D, Deng H, Wang D, Sun Y, et al. In-depth serum proteomics reveals biomarkers of psoriasis severity and response to traditional Chinese medicine. Theranostics. 2019;9(9):2475–2488. 31131048 10.7150/thno.31144 PMC6526001

[82] Yang N, Tian C, Lv Y, Hou J, Yang Z, Xiao X, Zhang Y. Novel primers for 16S rRNA gene-based archaeal and bacterial community analysis in oceanic trench sediments. Appl Microbiol Biotechnol. 2022;106(7):2795–2809. 35348850 10.1007/s00253-022-11893-3

[83] Wang P, Li J, Luo X, Ahmad M, Duan L, Yin L, Fang B, Li S, Yang Y, Jiang L, et al. Biogeographical distributions of nitrogen-cycling functional genes in a subtropical estuary. Funct Ecol. 2022;36(1):187–201.

[84] Martin M. CUTADAPT removes adapter sequences from high-throughput sequencing reads. EMBnet J. 2011;17(1):10–12.

[85] Edgar RC. UNOISE2: improved error-correction for Illumina 16S and ITS amplicon sequencing. bioRxiv. 2016. 10.1101/081257

[86] Bokulich NA, Kaehler BD, Rideout JR, Dillon M, Bolyen E, Knight R, Huttley GA, Gregory Caporaso J. Optimizing taxonomic classification of marker-gene amplicon sequences with QIIME 2’s q2-feature-classifier plugin. Microbiome. 2018;6(1): Article 90. 29773078 10.1186/s40168-018-0470-z PMC5956843

[87] Bolyen E, Rideout JR, Dillon MR, Bokulich NA, Abnet CC, Al-Ghalith GA, Alexander H, Alm EJ, Arumugam M, Asnicar F, et al. Reproducible, interactive, scalable and extensible microbiome data science using QIIME 2. Nat Biotechnol. 2019;37(8):852–857. 31341288 10.1038/s41587-019-0209-9 PMC7015180

[88] Luo X, Wang P, Li J, Ahmad M, Duan L, Yin L, Deng Q, Fang B, Li S, Li W. Viral community-wide auxiliary metabolic genes differ by lifestyles, habitats, and hosts. Microbiome. 2022;10(1): Article 190. 36333738 10.1186/s40168-022-01384-y PMC9636769

[89] Chen S, Zhou Y, Chen Y, Gu J. Fastp: An ultra-fast all-in-one FASTQ preprocessor. Bioinformatics. 2018;34(17):i884–i890. 30423086 10.1093/bioinformatics/bty560 PMC6129281

[90] Nurk S, Meleshko D, Korobeynikov A, Pevzner PA. metaSPAdes: A new versatile metagenomic assembler. Genome Res. 2017;27(5):824–834. 28298430 10.1101/gr.213959.116 PMC5411777

[91] Hyatt D, Chen G-L, LoCascio PF, Land ML, Larimer FW, Hauser LJ. Prodigal: Prokaryotic gene recognition and translation initiation site identification. BMC Bioinformatics. 2010;11(1): Article 119. 20211023 10.1186/1471-2105-11-119 PMC2848648

[92] Deng Z, Münch PC, Mreches R, McHardy AC. Rapid and accurate identification of ribosomal RNA sequences via deep learning. Nucleic Acids Res. 2022;50(10): Article e60. 35188571 10.1093/nar/gkac112 PMC9177968

[93] Langmead B, Salzberg SL. Fast gapped-read alignment with Bowtie 2. Nat Methods. 2012;9(4):357–359. 22388286 10.1038/nmeth.1923 PMC3322381

[94] Uritskiy GV, DiRuggiero J, Taylor J. MetaWRAP—A flexible pipeline for genome-resolved metagenomic data analysis. Microbiome. 2018;6(1): Article 158. 30219103 10.1186/s40168-018-0541-1 PMC6138922

[95] Parks DH, Imelfort M, Skennerton CT, Hugenholtz P, Tyson GW. CheckM: Assessing the quality of microbial genomes recovered from isolates, single cells, and metagenomes. Genome Res. 2015;25(7):1043–1055. 25977477 10.1101/gr.186072.114 PMC4484387

[96] Seemann T. Prokka: Rapid prokaryotic genome annotation. Bioinformatics. 2014;30(14):2068–2069. 24642063 10.1093/bioinformatics/btu153

[97] Li J, Sun W, Cao Y, Wu J, Duan L, Zhang M, Luo X, Deng Q, Peng Z, Mou X, et al. Increased temperature enhances microbial-mediated lignin decomposition in river sediment. Microbiome. 2025;13(1): Article 89. 40170118 10.1186/s40168-025-02076-z PMC11959967

[98] Buchfink B, Xie C, Huson DH. Fast and sensitive protein alignment using diamond. Nat Methods. 2015;12(1):59–60. 25402007 10.1038/nmeth.3176

[99] Zhu T, Zhu Y, Xuan Y, Gao H, Cai X, Piersma SR, Pham TV, Schelfhorst T, Haas RRGD, Bijnsdorp IV, et al. DPHL: A DIA pan-human protein mass spectrometry library for robust biomarker discovery. Genomics Proteomics Bioinformatics. 2020;18(2):104–119. 32795611 10.1016/j.gpb.2019.11.008 PMC7646093

[100] Lou R, Shui W. Acquisition and analysis of DIA-based proteomic data: A comprehensive survey in 2023. Mol Cell Proteomics. 2024;23(2): Article 100712. 38182042 10.1016/j.mcpro.2024.100712 PMC10847697

[101] Ren Y, Yu G, Shi C, Liu L, Guo Q, Han C, Zhang D, Zhang L, Liu B, Gao H, et al. Majorbio Cloud: A one-stop, comprehensive bioinformatic platform for multiomics analyses. iMeta. 2022;1(2): Article e12. 38868573 10.1002/imt2.12 PMC10989754

[102] Asnicar F, Thomas AM, Beghini F, Mengoni C, Manara S, Manghi P, Zhu Q, Bolzan M, Cumbo F, May U, et al. Precise phylogenetic analysis of microbial isolates and genomes from metagenomes using PhyloPhlAn 3.0. Nat Commun. 2020;11(1): Article 2500. 32427907 10.1038/s41467-020-16366-7 PMC7237447

[103] Letunic I, Bork P. Interactive Tree Of Life v2: Online annotation and display of phylogenetic trees made easy. Nucleic Acids Res. 2011;39:W475–W478. 21470960 10.1093/nar/gkr201 PMC3125724

