# Supplementary material for: Lignin Unlocks Stealth Carbon Sinks in Cold Seeps via Microbial Enzymatic Gatekeeping
Source: Research (Wash D C). 2025 Aug 25;8:0848. doi: 10.34133/research.0848 (PMC12377529; doi:10.34133/research.0848)
Supplement: Supplementary 1 — Figs. S1 to S5 Tables S1 to S10 [file research.0848.f1.zip › Supplementary.docx]

**Lignin unlocks stealth carbon sinks in cold seeps via microbial enzymatic gatekeeping**

**Fig. S1** TreeBar analysis based on Average Euclidean distances, illustrating the clustering relationships of samples from different layers over time (a); Analysis of core microbial groups showing the change in the number of core phyla with increasing cultivation time (b); Presentation of the predominant microbial community at class-level shared among samples at different time points (c).


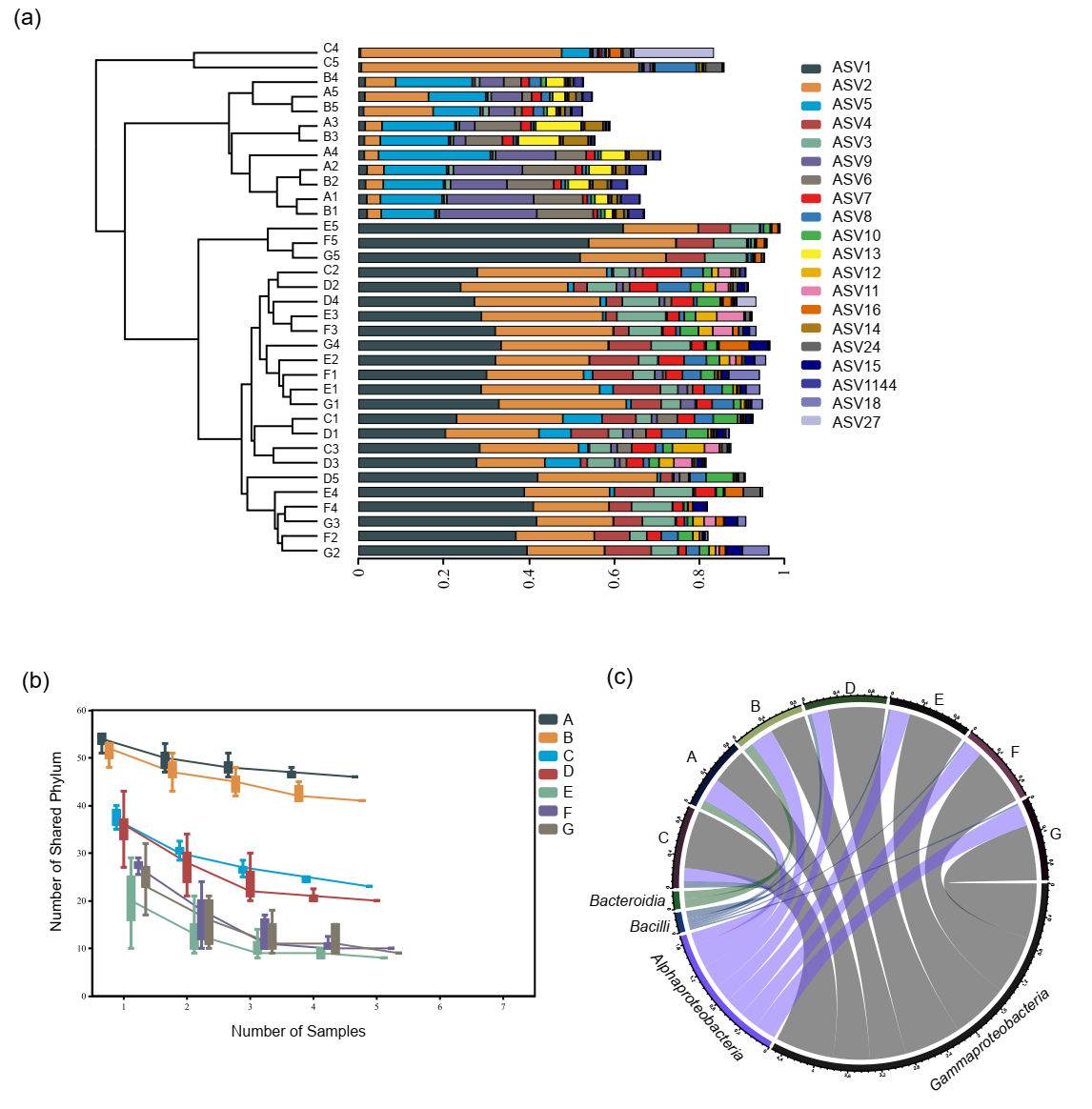


**Fig. S2 The degradation of lignin was observed before and after culture (90 days).**


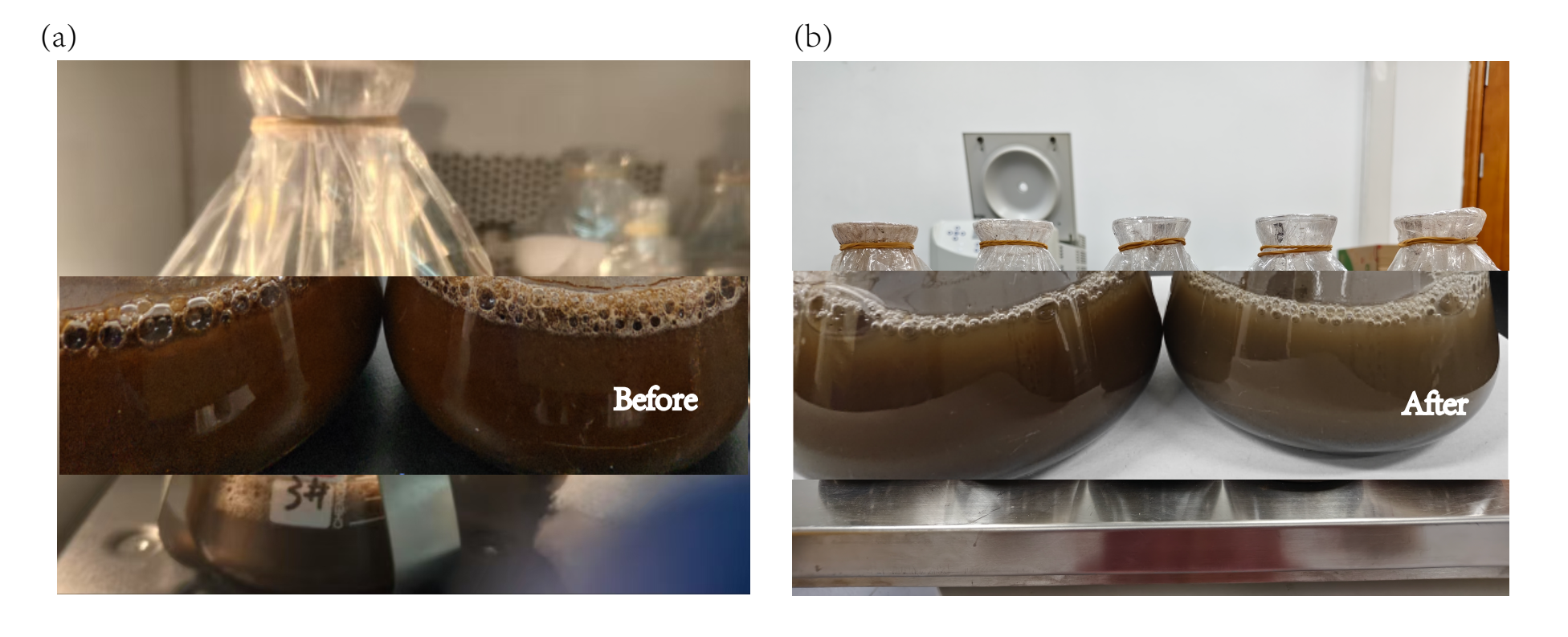


**Fig. S3 Summarizes the number of proteins involved in different metabolic processes as identified by metaproteomics.** Highlights the predominance of proteins associated with carbon metabolism and methane metabolism.


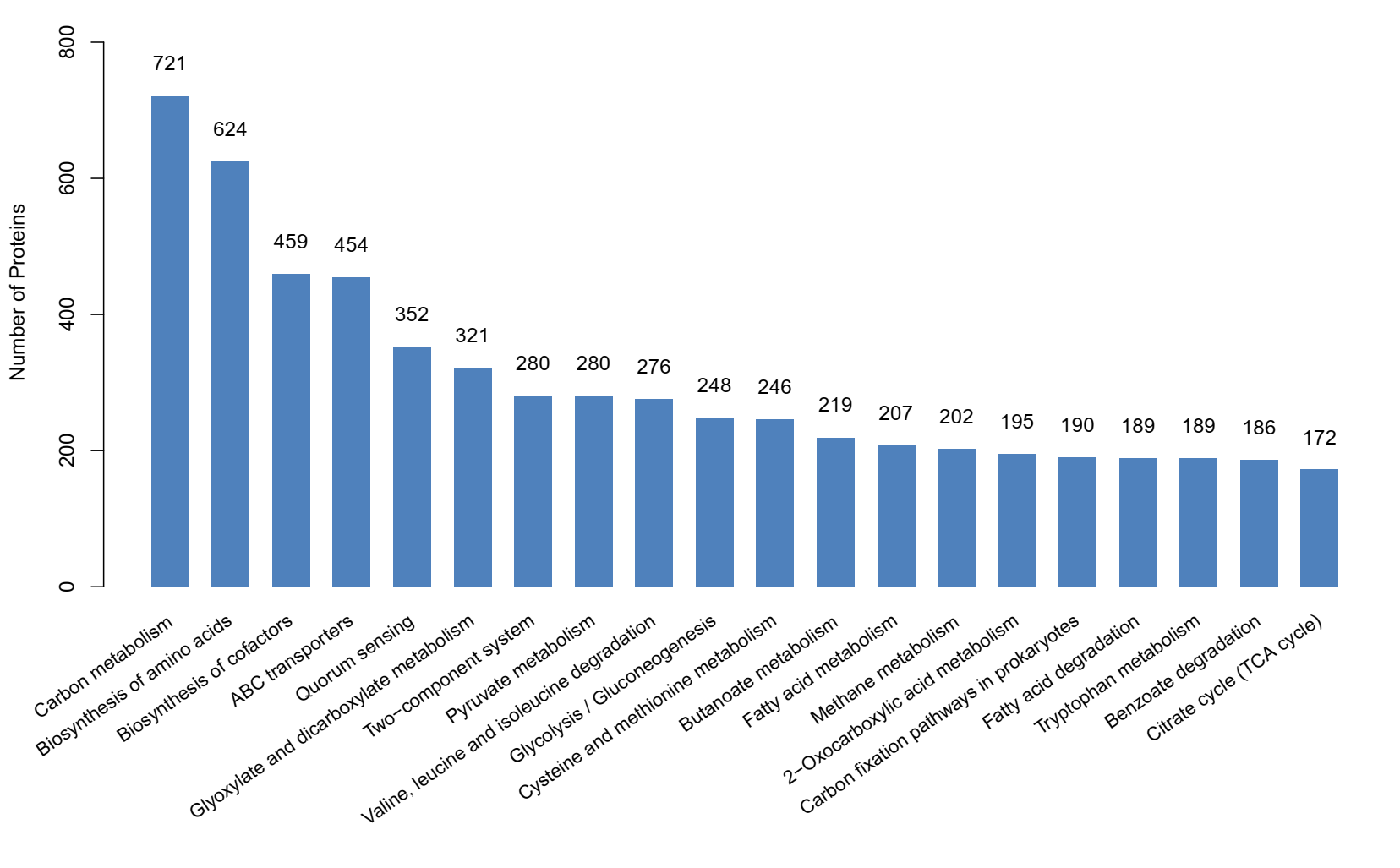


**Fig. S4 Methane production progresses based on the total protein.** The red boxes were the enzyme present in our sample, and the purple boxes were the enzyme that has been studied.


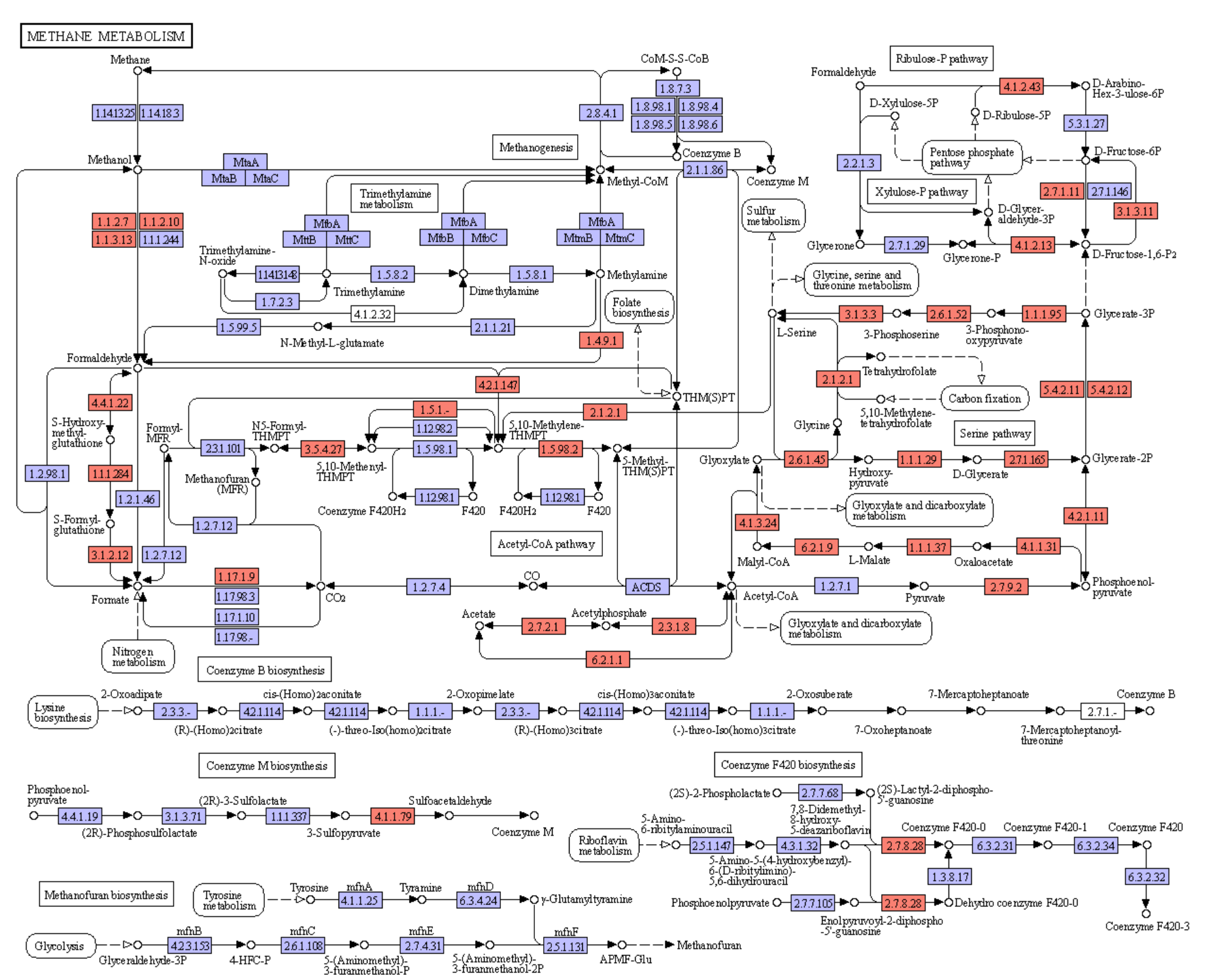


**
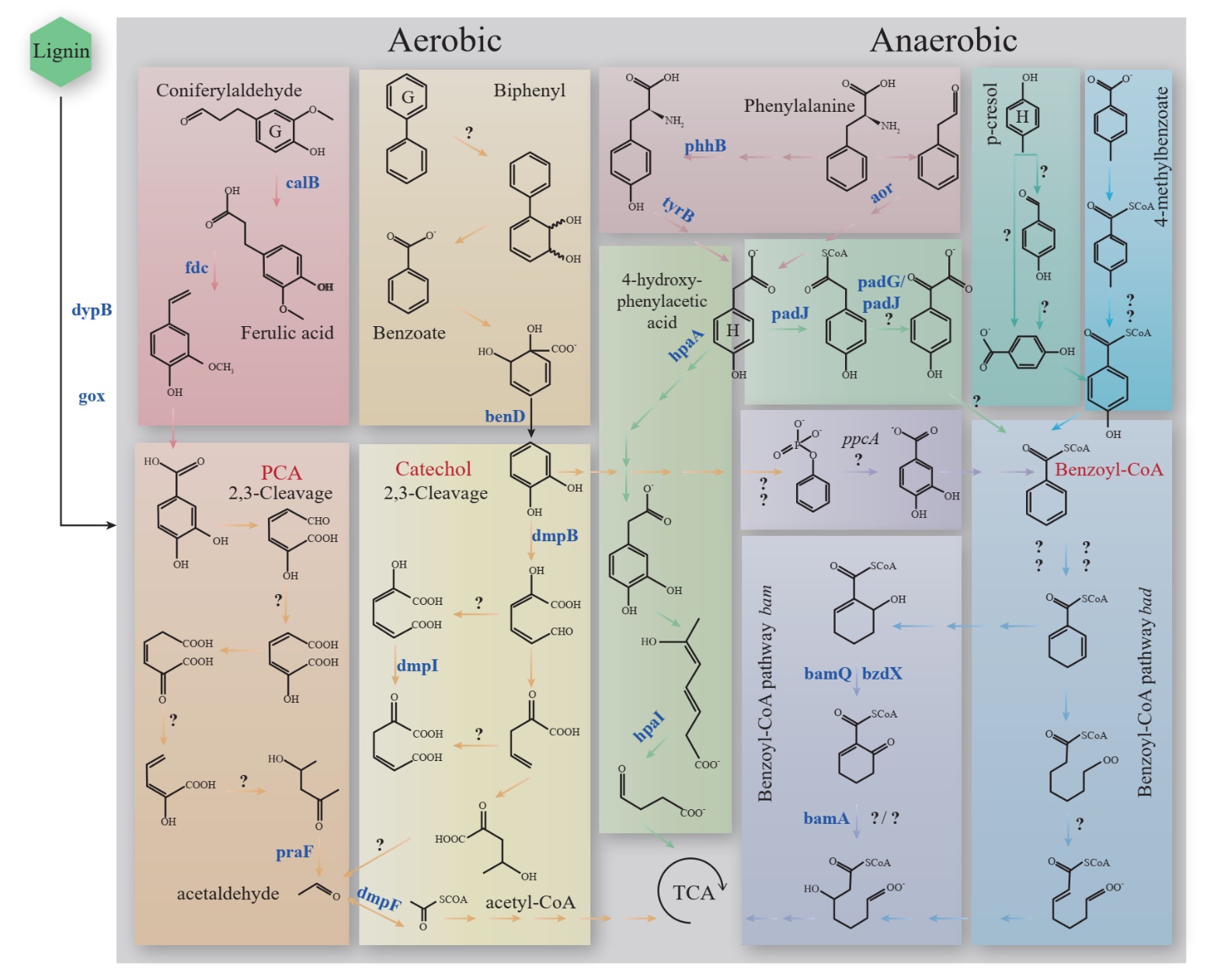
Fig.S5 Aerobic and anaerobic lignin degradation processes by microorganisms in Haima cold seep enriched sediments based on LCdb database.** Gene symbols indicate their presence in the sample; a question mark (?) denotes the absence of a gene.

**Tables title**

**Table S1**. Information of lignin-decomposition related gene including reactions steps, NCBI or UniProtKB accession numbers, E.C.Number, Auxiliary Activities family classification and Organism Classification.

**Table S2** The related ratios of lignin phenols were summarized, indicating demethyl/demethoxide degradation, and degradation process, vegetation source etc.

**Table S3** Summary information of the high-quality MAGs with lignin-decomposition related gene

**Table S4** The abundance of Cabon cycling genes in DNA or RNA based on RPKM value in different layers

**Table S5** The concentration of methane gas of lignin-enriched microbes from Haima Cold Seep Sediments in the headspace

**Table S6** Sampling and sequencing information for metagenomic and metatranscriptomic data used in this study, including geographic location of cold seeps, cold seep types, sample depths, and source references.

**Table S7** Taxonomic Profiles of Metagenome-Assembled Genomes from Globally representative Cold Seeps

**Table S8** Metagenome-Assembled Genomes containing Lignin Degradation Genes from Globally representative Cold Seeps

**Table S9** Metagenome-Contigs with Lignin Degradation Genes from enrichment cold seep sediment based on LCdb database

**Table S10** Metagenome-Assembled Genomes with Lignin Degradation Genes from Global representative Cold Seeps based on LCdb database
